# Characterization and Therapeutic Effect of a pH Stimuli Responsive Polymeric Nanoformulation for Controlled Drug Release

**DOI:** 10.3390/polym12061265

**Published:** 2020-06-01

**Authors:** Maria Victoria Cano-Cortes, Jose Antonio Laz-Ruiz, Juan Jose Diaz-Mochon, Rosario Maria Sanchez-Martin

**Affiliations:** 1 GENYO, Centre for Genomics and Oncological Research, Pfizer/University of Granada/Andalusian Regional Government, PTS Granada, Avda. Ilustración 114, 18016 Granada, Spain; victoria.cano@genyo.es (M.V.C.-C.); josealazr@go.ugr.es (J.A.L.-R.); 2 Department of Medicinal & Organic Chemistry, Excellence Research Unit of “Chemistry Applied to Biomedicine and the Environment”, Faculty of Pharmacy, University of Granada, Campus de Cartuja s/n, 18071 Granada, Spain; 3 Biosanitary Research Institute of Granada (ibs. GRANADA), University Hospital, Av. del Conocimiento, s/n, 18016 Granada, Spain

**Keywords:** covalent drug conjugation, therapeutic nanodevice, polymeric nanoparticles, cancer therapy, controlled drug delivery

## Abstract

Despite the large number of polymeric nanodelivery systems that have been recently developed, there is still room for improvement in terms of therapeutic efficiency. Most reported nanodevices for controlled release are based on drug encapsulation, which can lead to undesired drug leakage with a consequent reduction in efficacy and an increase in systemic toxicity. Herein, we present a strategy for covalent drug conjugation to the nanodevice to overcome this drawback. In particular, we characterize and evaluate an effective therapeutic polymeric PEGylated nanosystem for controlled pH-sensitive drug release on a breast cancer (MDA-MB-231) and two lung cancer (A549 and H520) cell lines. A significant reduction in the required drug dose to reach its half maximal inhibitory concentration (IC50 value) was achieved by conjugation of the drug to the nanoparticles, which leads to an improvement in the therapeutic index by increasing the efficiency. The genotoxic effect of this nanodevice in cancer cells was confirmed by nucleus histone H2AX specific immunostaining. In summary, we successfully characterized and validated a pH responsive therapeutic polymeric nanodevice in vitro for controlled anticancer drug release.

## 1. Introduction

Nanomedicine for cancer therapy has become a promising therapeutic approach to overcome the various limitations of conventional small molecule chemotherapeutics by improving drug internalization and selective intracellular accumulation in cancer cells, easing the toxicity to normal tissues [1,2]. Polymeric nanoparticles possess remarkable properties when compared to other colloidal systems such as (i) higher stability, particularly in body fluids; (ii) a larger contact area between the nanoparticle and the biological target; and (iii) a rapid adsorption rate and accumulation in the tumor cellular interstices due to the enhanced permeability and retention (EPR) effect [3,4]. Moreover, polymeric nanoparticle–drug conjugates present advantages when compared to polymer–drug conjugates, such as tunability and high and predefined drug loading based on efficient conjugation of the active agents to polymeric nanocarriers [5].

One of the main advantages offered by nanoparticles (NPs) is their ability to release drugs in a controlled manner [6]. This controlled release can be achieved by implementing a stimulus-sensitive approach involving a two-step process: first, the nanosystem is preferentially accumulated at the target site through the EPR effect; then, the drug-loaded nanoparticles are directly activated by an external (light, temperature, etc.) or internal (pH, enzymatic, redox, etc.) stimulus to produce the local release of the drug [7,8]. In particular, pH has been used for a long time as a critical feature for the differentiation between healthy tissues and abnormal tissues. Although fluctuations may occur, the pH in most solid tumors is between 6 and 7 [9]. This pH difference opened a new pathway for the release of tumor-specific drugs in tumors and simultaneously reduces undesirable effects in healthy tissues. Several examples of pH-sensitive nanodevices such as amorphous calcium carbonate–silica nanoparticles (core/shell), N- (2-hydroxypropyl) ethacrylamide (HPMA), dendrimers, and gold nanoparticles have been reported [10,11,12,13,14]. 

The chemotherapeutic drug doxorubicin (DOX) has been widely used in clinic settings for the treatment of different types of cancer. However, its toxicity to healthy tissue with effects such as cardiotoxicity and the development of resistance to multiple drugs during prolonged treatment have limited its therapeutic use [15]. Doxil^®^, the first nanopharmaceutical approved by the U.S. Food and Drug Administration (FDA) in 1995, takes advantage of the EPR effect and moves passively to the tumors where the encapsulated doxorubicin is released [16]. Recently, many nanotechnology-based drug delivery systems have been reported for the selective release of doxorubicin [17,18,19]. 

However, there is still room for improvement in terms of the therapeutic efficiency, as compared with free doxorubicin. Most of these nanodevices are based on drug encapsulation, which can lead to undesired drug leakage, causing loss of efficiency and systemic toxicity. This drawback can be overcome by covalent conjugation of the drug to the nanoparticle. 

We have previously reported the use of polystyrene-based nanoparticles for the efficient conjugation of bioactive molecules of different types, such as sensors, proteins, and nucleic acids. In addition, polystyrene nanoparticles have been implemented for imaging, biosensing, tracking cellular proliferation using fluorescent nanoparticles, metallofluorescent nanoparticles for multimodal applications, and in cellulo proteomics using drug-loaded fluorescent nanoparticles [20,21,22]. These polymeric particles are inherently attractive as a delivery system due to certain advantages, such as being easy to handle and robust, with a defined drug loading capacity, tunebility, and lack of toxicity. These nanosystems can achieve efficient delivery through a passive but rapid mechanism, without significant alterations involving cellular gene profiling or proteomics [23,24]. To overcome the limitations of current encapsulation-based polymeric nanodevices, we developed an efficient loading strategy based on the covalent conjugation of doxorubicin to cross-linked polystyrene nanoparticles for selective drug release. Herein, we characterize this PEGylated polystyrene nanodevice loaded covalently with doxorubicin in a pH labile linker controlled manner, and evaluate the therapeutic efficiency in several cancer cell lines in vitro. 

## 2. Materials and Methods

### 2.1. Materials

All solvents and chemicals were purchased from Sigma-Aldrich (Haverhill, United Kingdom). Dulbecco’s modified Eagle’s medium (DMEM), Roswell Park Memorial Institute medium (RPMI), L-glutamine, 1% penicillin/streptomycin, trypsin-EDTA, and fetal bovine serum (FBS) were purchased from Gibco (Thermo Fisher Scientific, Allschwil, Switzerland).

### 2.2. Synthesis of Aminomethyl Polystyrene Nanoparticles (NPs)

Aminomethyl NPs (NAKED-NPs (**1**)) were prepared by dispersion polymerization as previously described [25]. Briefly, polyvinylpyrrolidone (PVP) (Mw 29000) was dissolved in ethanol/water (86:14) and deoxygenated via argon bubbling. Azobisisobutyronitrile (AIBN) was dissolved in styrene (freshly washed) with 4-vinylbenzylamine hydrochloride (VBAH) and 2% divinylbenzene (DVB). The dispersion was deoxygenated via argon bubbling before addition to the PVP/ethanol solution. The mixture was stirred under argon for 30 min before heating to 65 °C for 15 h. Nanoparticles were obtained by centrifugation and washed with methanol (2×) and water (2×). Finally, the nanoparticles were stored in water at 4 °C. Particle size distribution: 460 nm (PDI = 0.042); amino quantification: 0.057 mmol g^−1^ of amino groups; Nº of particles per gram: 1.96 × 10^13^; solid content (SC): 2%.

### 2.3. PEGylation of NPs

A double PEGylation of aminomethyl NPs (**1**) was carried out. Briefly, aminomethyl NPs (**1**) were conditioned in N,N-Dimethylformamide (DMF). Separately, an Fmoc-4, 7, 10-trioxa-1, 13-tridecanediamine succinamic acid (Fmoc-PEG-COOH) spacer (MW = 542 g/mol) (15 eq) was dissolved in DMF, then Oxyma (15 eq) and N,N′-Diisopropylcarbodiimide (DIC) (15 eq) was added and mixed for 10 min at room temperature. Then, this mixture solution was added to NPs and mixed for 2 h at 60 °C. Subsequently, the NPs were washed by centrifugation (13,400 rpm, 3–10 min) with DMF, methanol, and water to obtain Fmoc-PEGylated NPs (**2**) (100% yield, 0.054 mmol g^−1^ of amino groups). Next, Fmoc deprotection was achieved by treating nanoparticles with 20% piperidine/DMF (3 × 20 min). PEGylated NPs (**3**) were obtained by centrifugation and subsequently washed with DMF, MeOH, and deionized water. Next, a second PEGylation and deprotection step was carried out to obtain the double-PEGylated NPs (**4**) (100% yield, 0.053 mmol g^−1^ of amino groups).

### 2.4. Preparation of pH Responsive Therapeutic Polymeric Nanodevice: DOX-NPs (**7**)

A solution of succinic anhydride (15 eq) and DIPEA (15 eq) in DMF was added to the double PEGylated NPs (**4**), sonicated, and mixed for 2 h at 60 °C. Then, carboxyl functionalized NPs (**5**) were activated with Oxyme (15 eq) and DIC (15 eq) for 4 h. Then, they were centrifuged and a solution of 55% v/v hydrazine (15 eq) in DMF was added and mixed at 25 °C for 15 h. Next, hydrazide-NPs (**6**) were washed and conditioned in PBS. Finally, doxorubicin (1 eq) was dissolved in pH 6 PBS and added to hydrazine-NPs (**6**) and mixed at 50 °C for 15 h to yield DOX-NPs (**7**) (CE = 97%, 5.2 × 10^−9^ nmol DOX per NP).

### 2.5. Characterization of DOX-NPs (**7**)

#### 2.5.1. Nanoparticle Size Distribution, Zeta Potential, and Morphology

Particle mean size, size distribution, and zeta potential of DOX-NPs (**7**) were determined by dynamic light scattering (DLS) and were measured on a Zetasizer Nano ZS ZEN 3500 (NanoMalvern Panalytical, UK) in biological grade water in a disposable cuvette for size measurements or in a transparent disposable cuvette for zeta potential measurements. The shape and morphology of the NPs were observed using a LIBRA 120 PLUS de Carl Zeiss SMT transmission electron microscopy (TEM, Oberkochen, Germany). The conjugation of DOX to NPs was checked by flow cytometry using FACSCanto II (Becton Dickinson & Co., New Yersey, USA) and Flowjo^®^ 10 software for data analysis.

#### 2.5.2. Determination of Nanoparticle Concentration by Spectrophotometry

The concentration of nanoparticles per microliter (NPs/μL) was determined by a standard spectrophotometric method described previously by our group [26]. Briefly, a measurement of the turbidity optical density at 600 nm was performed for each of the preparations based on nephelometric principals. In this way, a calibration standard curve was obtained for aminomethyl NPs (**1**) using a set of concentrations (Supplementary Figure S1, see Supplementary Material). Calibration curves fitted linear regression models by which the number of NPs/μL corresponding to one unit of OD600 for each size could be determined. Thus, these curves using known concentrations of aminomethyl NPs (**1**) allowed us to estimate the number of NPs in the final batches, even after multiple handling procedures, by OD600 measurements of 1 μL of each preparation (Supplementary Figure S2, see Supplementary Material). This spectrophotometric method was used to calculate number of NPs/μL in all the preparations used in this study.

#### 2.5.3. Amino Quantification of Nanoparticles

To determine the capacity of conjugation of each batch of nanoparticles, the amount of reactive amino groups on the nanoparticle surface was calculated by the conjugation of a glycine with amino group protected with 9H-fluoren-9-yl-methoxycarbonyl (Fmoc) (Fmoc-Gly-OH) and quantification using the Fmoc test. The amount of released piperidine-dibenzofulvene adduct was quantified spectrophotometrically by measuring the absorbance of the solution at 302 nm using UV–vis light [27].

The amino quantification of the nanoparticles was calculated according to the equation

(1) $${\mathrm{Amino}\;\mathrm{quantification}\;(\text{µ}\mathrm{mol}\;\times \;g\;}^{-1})=\frac{{(A}_{302}\;\times \;V)}{{(\varepsilon }_{302}\;\times \;m\;\times \;d)}{\;\times \;1\;\times \;10}^{6}$$

where A302 is the absorbance of the supernatant measured at 302 nm, V is the volume of the measured solution (mL), ε302 is the molar extinction coefficient at 302 nm (7800 M^−1^ cm^−1^), d is the diameter of the cuvette (1 cm), and m is the mass of the NPs analyzed (mg).

#### 2.5.4. Evaluation of Drug-Loading Efficiency

To determine the amount of DOX loaded on the nanoparticle surface (loading capacity, LC) and to evaluate the efficiency of the conjugation process (conjugation efficiency, CE), the concentration of free DOX in the supernatant obtained after the centrifugation of NPs was measured by UV spectroscopy at 480 nm. A calibration curve, with the lineal ratio between the optic density of DOX and its concentration, was generated (Supplementary Figure S3, see Supplementary Material). Then, the DOX loading capacity (LC) and DOX conjugation efficiency (CE %) were calculated according to the following formulas

(2) $$LC=\frac{\left[\mathrm{DOX}\;\mathrm{conjugated}\;\mathrm{on}\;\mathrm{surface}\;\mathrm{of}\;\mathrm{nanoparticle}\right]}{\;\mathrm{Number}\;\mathrm{of}\;\mathrm{NPs}}\times N_{A}$$

where *N*_A_ is Avogadro’s number.

(3) $$CE\left(\%\right)=\frac{\left[\mathrm{DOX}\;\mathrm{conjugated}\;\mathrm{on}\;\mathrm{surface}\;\mathrm{of}\;\mathrm{nanoparticle}\right]}{\mathrm{Total}\;\mathrm{concentration}\;\mathrm{of}\;\mathrm{DOX}\;\mathrm{added}}\times 100$$

#### 2.5.5. Drug Release Profile

The drug release profile of the NPs was determined by analyzing the efficiency of the hydrolysis of the hydrazone bond of the DOX-NPs (**7**) samples at acidic pH. Briefly, 4.80 × 10^10^ DOX-NPs (**7**) were incubated in a phosphate solution at pH 6 and at pH 7.4 for 168 h (7 days) in an incubator at 37 °C. The supernatants were collected by centrifuging each sample at t = 1, 3, 6, 24, 48, 72, and 168 h, and they were analyzed using high performance liquid chromatography (HPLC) (Agilent 1200 series HPLC system). A calibration curve of Doxorubicin was generated using standard samples (Supplementary Figure S4, see Supplementary Material). Cumulative release was determined using the equation

(4) $$\left(\%\right)=\frac{D_{t}}{D_{T}}\times 100$$

where *D*_t_ is the concentration of DOX released from the DOX-NPs (**7**) at time t and *D*_T_ is the concentration of DOX-loaded onto the DOX-NPs (**7**).

### 2.6. Cell Cultures

Cell lines were provided by the cell bank of the CIC of the University of Granada. Three different cell lines were used in this study. The non-small cell lung cancer cell line H520 was cultured in RPMI supplemented with 10% (v/v) FBS, 1% L-Glutamine, and 1% penicillin/Streptomycin. The A549 (lung cancer) cell line and MDA MB 231 (human breast cancer) cell line were cultured in DMEM supplemented with 10% (v/v) FBS, 1% L-Glutamine, and 1% Penicillin/Streptomycin. All cell lines were grown in a humidified incubator at 5% CO_2_ and 37 °C. All cell lines tested negative for mycoplasma infection.

### 2.7. Nanofection of Cancer Cell Lines

A549, H520, and MDA MB 231 cell lines were incubated with DOX-NPs (**7**) (NPs/cells) for the established incubation times in a humidified incubator at 5% CO_2_ and 37 °C. Untreated cells and cells treated with NAKED-NPs (**1**) were used as control. After the incubation time, cells were detached and washed with PBS 1×. Then, samples were fixed in 2% paraformaldehyde and analyzed via flow cytometry using a FACSCanto II flow cytometer and confocal microscopy (see details of the nanofection protocol in **Supplementary Material**).

### 2.8. Cell Viability

The cellular cytotoxicity of the DOX-NPs (**7**) was determined using the resazurin cell viability assay (Sigma Aldrich). This quantitative fluorometric method is based on the ability of living cells to convert resazurin (a redox dye) into a fluorescent end product, which is measured at 570 nm directly from 96-well plates. The cells were seeded in a 96-well plate at pre-optimized concentrations, depending on the assay performed. The results were evaluated according to the manufacturer’s protocol, and the amount of fluorescence obtained was proportional to the number of viable cells. Viability was expressed with respect to the percentage of untreated cells (100%). Control wells were included in each plate to measure the fluorescence of the culture medium with nanoparticles added in the absence of cells.

### 2.9. Determination of DNA Damage in Cancer Cells by Immunostaining of Phospho-H2A.X Foci

A549, H520, and MDA MB 231 cells were cultured in DMEM supplemented with 10% FBS, L-glutamine, and penicillin/streptomycin on coverslips in 24-well plates. Cells were incubated with 5000 DOX-NPs (**7**) per cell. After 24 h of incubation, the media was replaced with fresh full DMEM. One hour after the change of the medium, the cells were fixed with 4% PFA for 10 min at room temperature. After fixation, the cells were washed with PBS and incubated with blocking buffer containing 5% BSA and 0.3% Triton X-100 in PBS for 1 h at room temperature. Then, cells were incubated with a 1:500 solution of primary antiphospho-H2A.X antibodies in blocking buffer (Cell Signaling, 20E3, 1:500) at +4 °C overnight (300 μL/well). The next day, cells were washed with PBS and stained with a 1:1000 solution of secondary Alexa Fluor 488 conjugated antibodies (Invitrogen, A-11034) for 1 h at room temperature. After washing with PBS, the preparations were mounted with mounting medium including antifade and DAPI (Invitrogen) [28].

### 2.10. Statistical Analysis

The data are presented as the mean ± the standard deviation in the error bars. The sample size (n) indicates the experimental repeats of a single representative experiment (3, unless otherwise specified). The results of the experiments were validated by independent repetitions. Graphs and statistical difference data were made with GraphPad Prism 6.0 (GraphPad Software Inc.). Statistical significance was determined using Student’s *t*-test in paired groups of samples with a known median and two-way analysis of variance (ANOVA,), and with a Bonferroni’s post-hoc test when comparing more than two groups of samples. A *p*-value of ≤0.05 was considered significant.

## 3. Results and Discussion

### 3.1. Preparation of DOX-NPs (**7**)

**Scheme 1:**
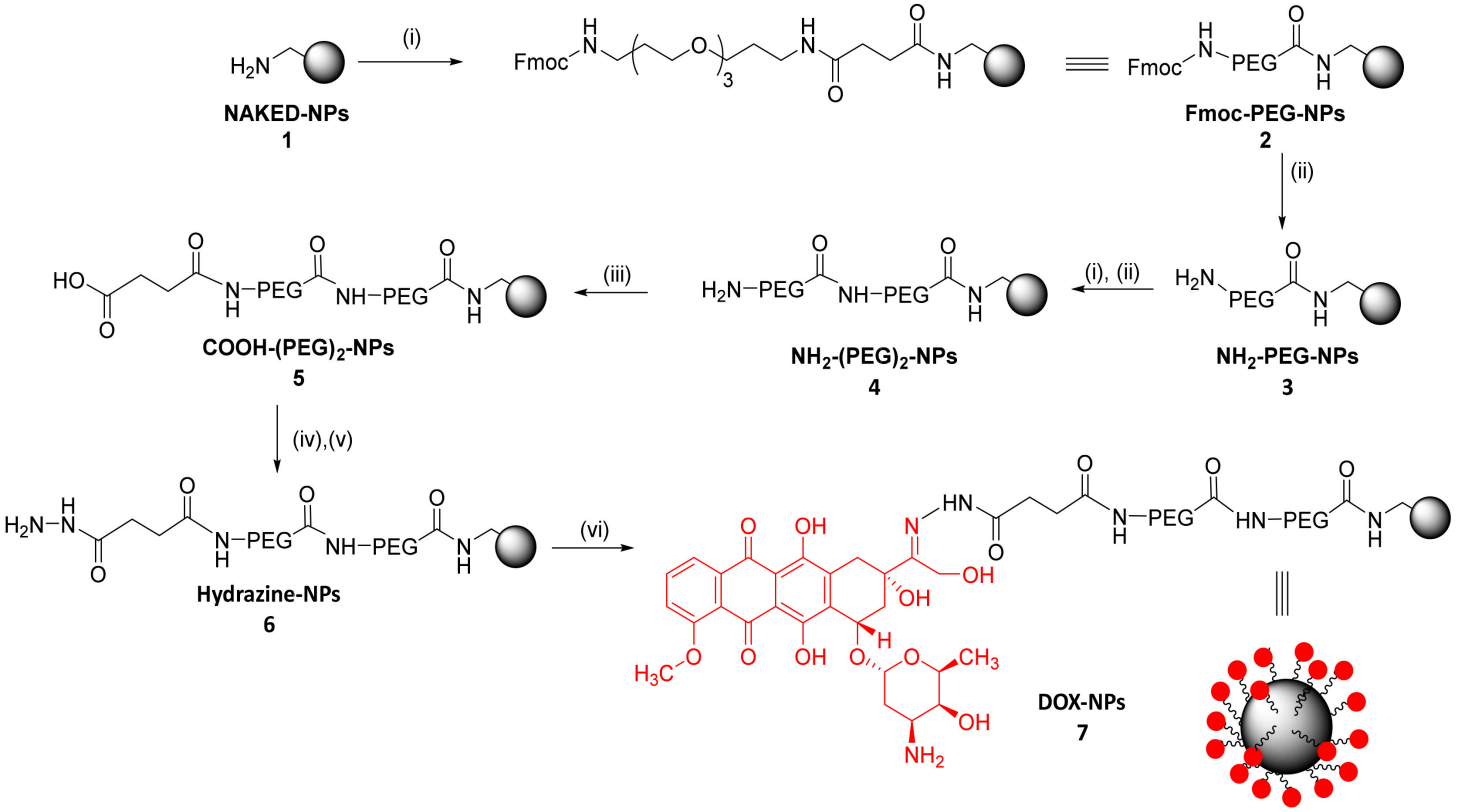
Preparation of the pH responsive therapeutic polymeric nanodevice DOX-NPs (**7**). Reagents and conditions: (i) Fmoc-PEG-COOH (15 eq), Oxyma (15 eq), DIC (15 eq), DMF, 2 h, 60 °C; (ii) 20% piperidine/DMF, 3 × 20 min; (iii) Succinic anhydride (15 eq), DIPEA (15 eq), 2 h, 60 °C; (iv) Oxyma (15 eq), DIC (15 eq), 2 h, 25 °C; (v) Hydrated hydrazine 55% v/v (15 eq), 15 h, 25 °C; (vi) Doxorubicin (1 eq), PBS, 15 h, 50 °C.

Following a previously described protocol, a 500 nm monodisperse population of polystyrene amino-functionalized NPs (**1**) with 2% divinylbenzene (DVB) crosslinking were synthesized by dispersion polymerization (using 4-vinylbenzylamine hydrochloride– VBAH as the monomer to functionalize the nanoparticle with the amino groups) [25]. Following an Fmoc solid phase protocol, aminomethyl NPs (**1**) were double PEGylated (**4**) (100% yield). The density of PEGylation was calculated based on the amount of amino groups on the nanoparticle, this value being 3.57 × 10^−9^ nmoles PEG/NP. The PEGylation increases the biocompatibility of the NPs, thereby facilitating their transport across cell membranes. It also reduces unfavorable interactions between NPs and the bioactive cargoes. Then, drug loading was carried out. The drug of choice was DOX due to its broad clinical use and it being one of the most effective chemotherapeutics for treating cancers, such as lung, breast, gastric, sarcoma, and pediatric cancers [29,30,31]. The designed strategy for the conjugation of DOX was via hydrazone bond. For this purpose, carboxylated NPs (**5**) were prepared using succinic anhydride; then, hydrazine functionalized NPs (**6**) were prepared, and the selective conjugation to the keto group in position C-13 of doxorubicin was carried out to yield DOX-NPs (**7**) (Scheme 1).

#### Characterization of Drug-Loaded Nanoparticles (DOX-NPs) (**7**)

**Figure 1:**
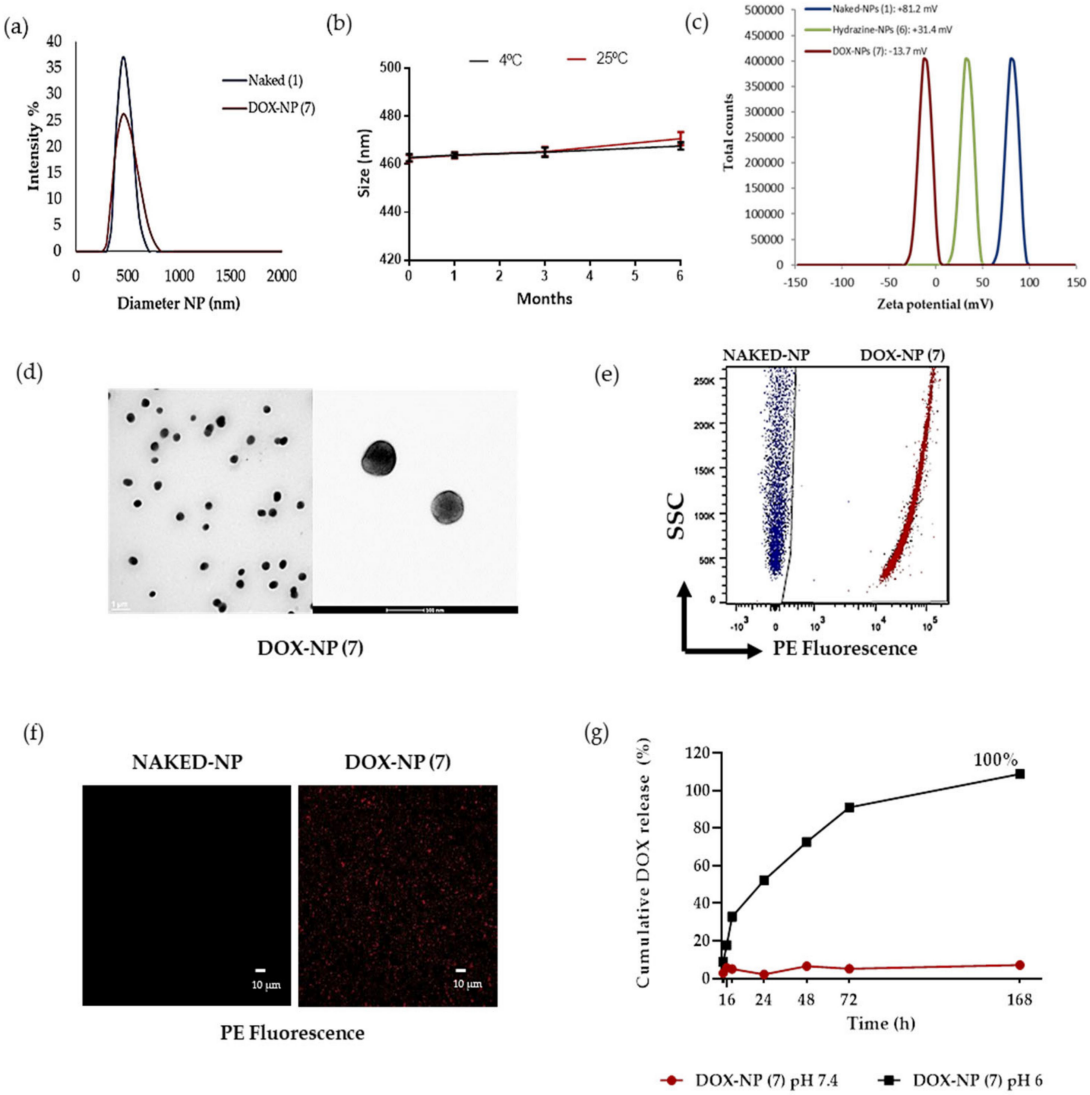
Characterization of the DOX-NPs (**7**). (**a**) Hydrodynamic diameter of DOX-NPs (**7**) versus Naked-NPs (**1**); (**b**) Stability of DOX-NPs (**7**) for six months at 4 °C and 25 °C; (**c**) Values of the zeta potential of DOX-NPs (**7**) (red) versus Hydrazine-NPs (**6**) (green) and NAKED-NPs (**1**) (blue); (**d**) Transmission electron microscopy (TEM) analysis; (**e**) Dot plots representative of flow cytometry of NAKED-NPs (**1**) (blue) and DOX-NPs (**7**) (red); (**f**) Images of confocal laser microscopy of naked nanoparticles and DOX-NPs (**7**). Images with an increase of 63x show the signal of red channel. Scale bar, 10 μmsignal of red channel. Scale bar, 10 μm. (**g**) Cumulative DOX Release. DOX-NPs (**7**) were incubated in phosphate solution at pH 6 and PBS at pH 7.4 for 168 h at 37 °C.The results are expressed with the values of the mean ± SEM.

The size distribution and zeta potential of the nanoparticles loaded with doxorubicin, DOX-NPs (**7**), together with amino functionalized nanoparticles, NAKED-NPs (**1**) were determined quantitatively by DLS (Figure 1a,c). The results obtained show a hydrodynamic diameter of 464.2 ± 0.9 nm with a PDI of 0.047, demonstrating that nanoparticle population was monodisperse (Figure 1a). The size was corroborated by TEM analysis (Figure 1d). The zeta potential value of DOX-NPs (**7**) was slightly negative (−13.7 mV ± 0.9) in water, as compared to NAKED-NPs (**1**), which was +81.2 mV ± 0.8, and Hydrazine-NPs (**6**)) which was +31.4 mV ± 0.3. (Figure 1c)). Keeping in mind that it has been described that to maintain the stability through electrostatic repulsion, zeta potentials of a particle should be above −20 mV, it could be predicted that the stability of these particles could be compromised. However, we will like to highlight that these particles are stable. A stability assay has been runned (see Figure 1b) and DOX-NPs (**7**) were stable for six months at 4 °C and 25 °C. As the nature and number of molecules coated on the surfaces can affect the stability of polymeric particles, we could suggest that the balance between the loaded drug and the PEGylation on the surface has been a positive effect on the stability of these nanoparticles.

The effect of storage conditions on the long-term stability of the NPs was investigated. The stability ofDOX-NPs (**7**) was evaluated by DLS (Figure 1b). The size of these nanodevices was measured after 1, 3, and 6 months at 4 °C and 25 °C, showing a constant size distribution (Figure 1a,b). DLS measurements revealed that the long-term stability of these nanoparticles was not influenced by storage temperature conditions. These results confirm that these particles are stable for a long time allowing them to be stored. This is a key property for further translation of this nanodevice.

On the basis of the fluorescence properties of DOX (λex = 470 nm, λem = 560 nm), the efficiency of drug conjugation onto the nanoparticles was monitored easily using fluorescence-based techniques such as flow cytometry and microscopy. Figure 1e shows a representative plot of the obtained results by flow cytometry analysis. An increase in fluorescence of the DOX-NPs (**7**) conjugates with respect to unloaded nanoparticles (NAKED-NPs (**1**)) can be observed. This result was corroborated by confocal microscopy (Figure 1f).

In order to evaluate the efficiency of the conjugation of doxorubicin to DOX-NPs (**7**), a spectrophotometric quantification of the remained unconjugated drug in the supernatant of the reaction was measured by UV spectroscopy (A480 nm). A calibration curve, with a lineal ratio between the optic density of doxorubicin and its concentration using a set of standard samples, was generated (Supplementary Figure S3, see Supplementary Material). To determine the value of loading capacity (LC), the amount of conjugated drug with respect to the number of nanoparticles was considered instead of the nanoparticle weight (as frequently reported) [31]. For this approach, an accurate spectrophotometric method to determine the number of nanoparticles per volume previously developed by our team was carried out [26]. The concentration of nanoparticles DOX-NPs (**7**) was estimated as 3.72 × 10^8^ NPs/μL. The drug loading capacity is related to the number of nanoparticles, thereafter, the loading capacity per nanoparticle can be calculated. A loading capacity of 5.2 × 10^−9^ nmol DOX per NP was estimated. This parameter provides the value of drug dose with precision and accuracy; this fact being of extreme relevance for the clinical translation of nanomedicine. Taking into account the drug conjugated with respect to the total amount of the drug, the conjugation efficiency (CE) was 89%. These results shows the high efficiency of this nanodevice compared to previously reported therapeutic nanodevices based on drug encapsulation [18].

A major deficiency of the sustained release system is that the release is not specific. To ensure the release of the drug at the target site, to avoid a non-specific release, a pH-sensitive stimuli release strategy was implemented. The hydrazone is used in our approach as a cleavable bond that responds to stimuli depending on the pH; very useful in a tumor microenvironment that has a slightly acidic pH [32]. In order to release the drug in acidic conditions, doxorubicin was covalently conjugated to nanoparticles by a hydrazone bond sensitive to pH 6. Therefore, it is necessary to determine the pH-responsive drug release to evaluate this nanodevice. A high performance liquid chromatography (HPLC) assay was conducted to monitor the release process of doxorubicin in vitro. Release profiles were obtained by comparing the percentage of the released drug with respect to the amount of doxorubicin conjugated to the DOX-NPs (**7**) for seven days at pH 6 and pH 7.4 by HPLC analysis. As shown in Figure 1g (blackg line), in an acidic environment (pH 6 PBS), pH-sensitive cleavage of the hydrazone linker resulted in the exponential sustained release of the drug, and an accumulative release was obtained for up to one week (168 h). These results were expected as the hydrazone bond is sensitive to pH 6, and the drug is consequently released from the nanoparticles. A burst release was achieved within 24 h of incubation at pH 6 reaching a release rate of 52% ± 0.26. Additionally, a sustained release occurs for up to 168 h, achieving a maximum release value of 100%. Furthermore, in a simulated neutral physiological environment (pH 7.4 PBS), the amount of doxorubicin released from the nanodevice did not reach 10%, which indicated that the drug remained attached to the nanoparticles (Figure 1g, red line). This result indicated a remarkable stability and selectivity of the DOX-NPs (**7**). Therefore, this ability of DOX-NP (**7**) to release doxorubicin in a sustained manner in acidic tissues, such as tumoral tissues, could be a very beneficial feature to prolong and improve the therapeutic efficacy of NPs in the tumor. Consequently, the pH value of the medium has a clear effect on the release efficiency of doxorubicin, which validates the drug release strategy designed for this approach.

### 3.2. Evaluation of the Efficiency of Cellular Uptake of DOX-NP (**7**)

**Figure 2:**
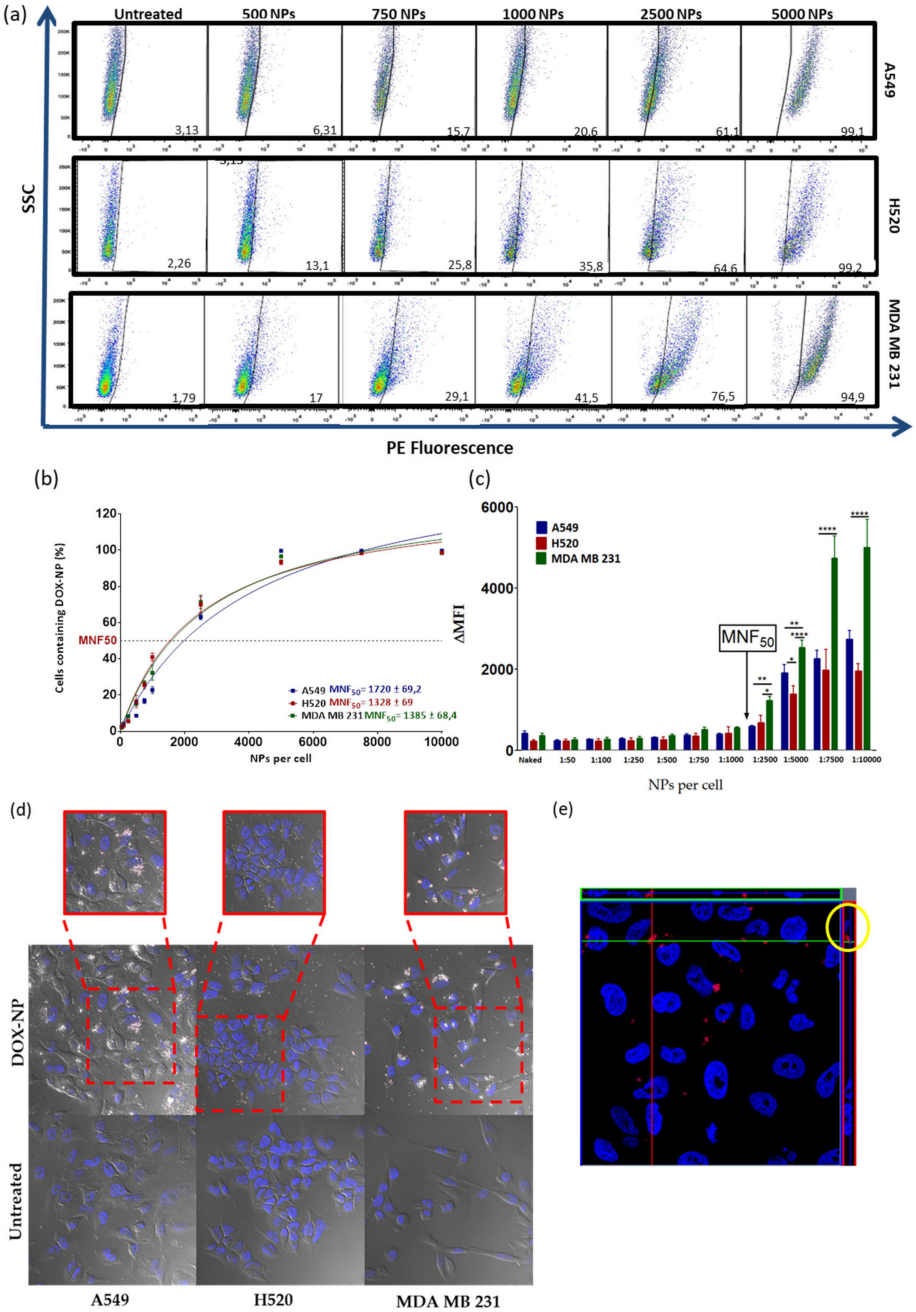
Evaluation of the cellular nanofection of DOX-NPs (**7**) in the cell lines A549, H520, and MDA MB 231 by flow cytometry. (**a**) Dot plots representative of flow cytometry obtained by incubating different concentrations of NPs for 24 h in the cell lines. (**b**) Percentage of cells containing the different concentrations of DOX-NPs (**7**). The data (mean ± SEM) are represented with a hyperbola equation model showing the different degrees of cellular internalization according to the number of DOX-NPs (**7**). (**c**) Representation of bars to compare ΔMFI between cell lines: A549 (blue), H520 (red), and MDA MB 231 (green). The arrow indicates the MNF50. The experiments were carried out in triplicate and the results are expressed with the values of the mean ± SEM. The statistical significance was determined by the analysis of the variance of a factor (ANOVA) using the Bonferroni multiple comparison (* *p*-value < 0.05, ** *p*-value < 0.001, **** *p*-value < 0.0001). MNF50: Multiplicity of nanofection 50. (**d**) Evaluation of uptake cellular of DOX-NPs (**7**) in the cell lines analyzed by confocal microscopy. The cells were incubated for 24 h with 2000 NPs/cell. The untreated cells were used as a negative control. Images with an increase of 63× show a composition of the three channels used: DIC; blue, DAPI for the nucleus; and red for DOX-NPs (**7**). (**e**) Orthogonal view (xy, xz, and yz) of the confocal microscope images showing the intersection planes at the position of the cross-line. Maximum intensity projection of the z-stack from blue (DAPI, nuclei) and red (DOX-NPs (**7**)) in the A549 cell line is displayed.

To investigate the efficiency of nanofection (cellular uptake and internalization) of DOX-NPs (**7**),) three cancer cell lines were chosen: two from lung cancer (A549 and H520) and one from triple negative breast cancer (MDA MB 231). The selection of these cell lines was based on the fact that doxorubicin is routinely used as a first-line chemotherapeutic agent in the treatment of these types of cancer [29,30,31]. For this purpose, the number of nanoparticles per cell needed to cause 50% of the cells to be nanofected (MNF50 index) was calculated in order to quantify the nanofection capacity of DOX-NPs (**7**)) following a protocol previously reported by us [26]. Nanofection efficiency of the DOX-NPs (**7**) in these three cell lines was quantitatively determined by flow cytometry based on the intrinsic red fluorescence of DOX. To this end, different concentrations of these nanoconjugates were incubated for 24 h, in an increasing gradient of concentration with between 50 and 10,000 NPs added per cell. After the incubation time, the cells were washed, trypsinized, and analyzed by flow cytometry. The results obtained on the cellular nanofection of DOX-NPs (**7**) revealed that they are internalized efficiently by the cancer cells studied. As shown in Figure 2a, a concentration-dependent effect on uptake efficiency was observed in the three cell lines. The analysis of the data showed that these three cell lines demonstrated a very similar MNF50 index, with values of 1720 ± 69.2 in the A549, 1328 ± 69 in the H520, and 1385 ± 68.4 for the MDA MB 231 cells (Figure 2b). However, the analysis of the increase in the median fluorescence (ΔMFI) indicated that, although the MNF50 was similar in the three cell lines and the internalization capacity gradually increased as the number of DOX-NPs (**7**) increased, starting at 2500 NPs/cell, the behavior of the lung cancer lines became different from that of the breast cancer cell line; the fluorescence intensity in the MDA MB 231 cells doubled by increasing the concentration range until reaching the saturation level. While the internalization ofDOX-NPs (**7**) in the lung cells lines demonstrated a similar behavior, it was not the same, since the cellular entry was higher in the A549 without reaching the saturation point in both cases (Figure 2c). The cellular uptake of DOX-NPs (**7**) was also verified by confocal fluorescence microscopy. Figure 2d shows that cell internalization occurred efficiently in the three cell lines tested, confirming the cytoplasmic localization of DOX-NPs (**7**). Furthermore, to corroborate the intracellular location of DOX-NPs (**7**) and to discard any adsorption of these NPs onto the cell surface, a confocal microscopy analysis was carried out. Confocal microscopy image of A549 cells treated with DOX-NPs (**7**) showed that the location of these nanoparticles was intracellular (Figure 2e).

### 3.3. Evaluation of the Therapeutic Capacity of the DOX-NP (**7**)

**Figure 3:**
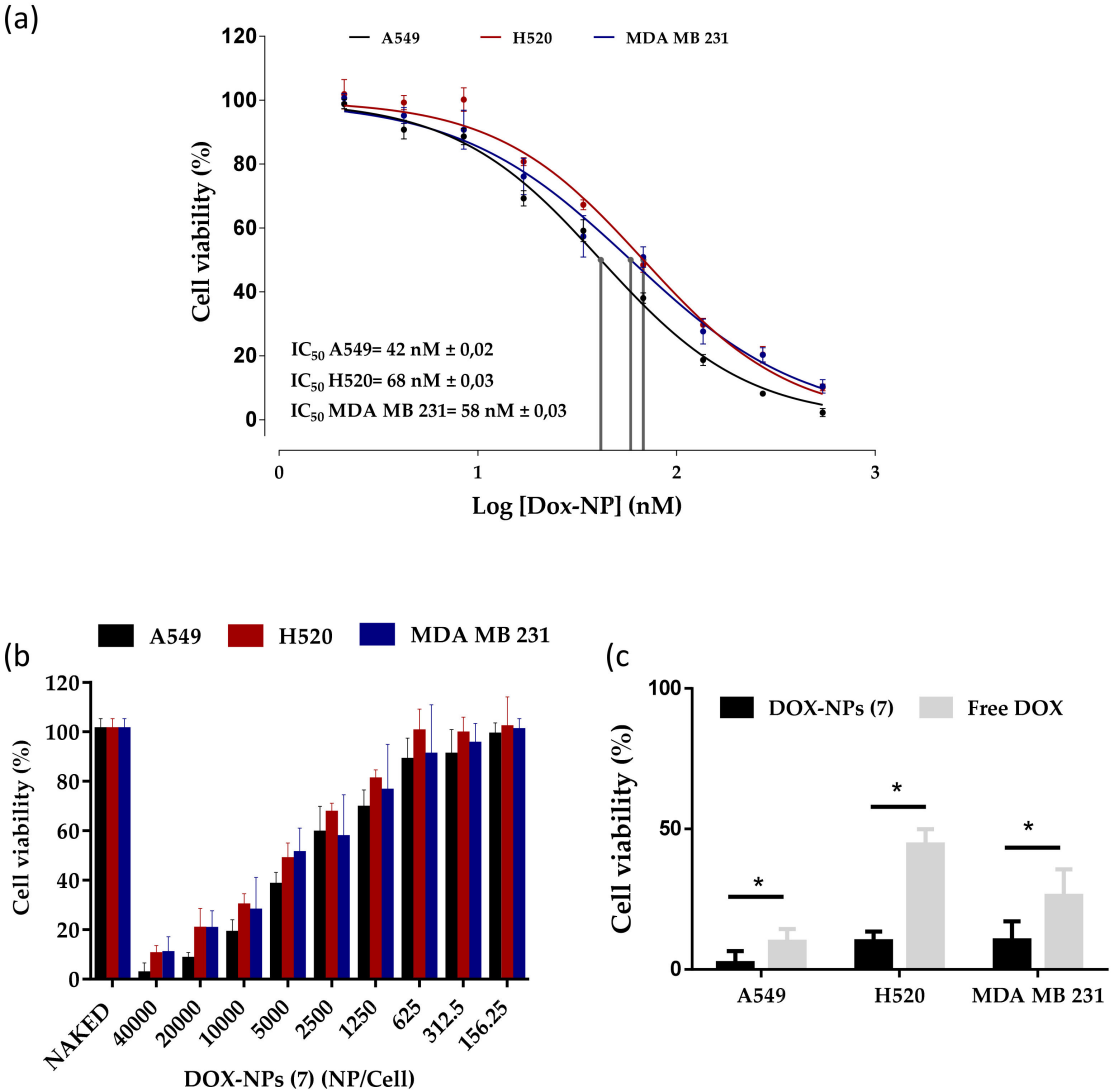
Effect of DOX-NPs (**7**) on cell viability. (**a**) Dose–response curves (percentage of cell viability versus concentration) of the treatment of A549, H520, and MDA-MB-231 cancer cells with DOX-NPs (**7**) represented in nM. The IC50 value was determined using the logarithm (inhibitor) versus normalized response: variable slope using the GraphPad software. (**b**) Bar graph showing cell viability of these three cell lines treated with DOX-NPs (**7**) and NAKED-NPs (**1**). (**c**) Comparison of therapeutic effect of DOX-NPs (**7**) compared to corresponding concentration of free doxorubicin (40,000 NPs/cell, 500 nM). Statistical significance was determined by Student’s *t*-test (* *p*-value < 0.0001). The viability data represent the mean ± SEM of the results of three independent experiments with six points each.

In order to verify the enhanced anticancer effect of doxorubicin conjugated to DOX-NPs (**7**), the proliferation inhibition was tested by measuring the cell-mediated reduction of sodium resazurin, a standard colorimetric and quantitative method that determines the cell viability on the cell lines studied [33]. A549, H520, and MDA-MB-231 cells were treated with a range of different NPs concentrations for 96 h. Free DOX as well as nanoparticles without the drug loaded (NAKED-NPs (**1**)) were use as positive and negative controls, respectively. As shown in Figure 3a,b, the cell proliferation inhibition efficacy of DOX-NPs (**7**) exhibited a strongly dose-dependent pattern after culture for 96 h. It is important to remark that the naked NPs (**1**) without doxorubicin have no effect on the cell viability of the three cells lines (Figure 3b). Half the maximal inhibitory concentration (IC50) was determined (Figure 3a). The IC50 value of DOX-NPs (**7**) was calculated to be 42 nM in the A549 cells, 68 nM in the H520 cells, and 58 nM in the MDA MB 231 cells (Figure 3a), which correspond to 3061, 5012, and 4325 NPs added per cell, respectively (Supplementary Figure S5, see Supplementary Material). DOX-NPs (**7**) were found to be considerably more efficient than free DOX on an equimolar basis (IC50 = 100 nM in A549, 186 nM in H520, and 120 nM in MDA MB 231 cells) (Supplementary Figure S6, see Supplementary Material). These results are in agreement with previously reported studies with free doxorubicin [13,14]. These values indicated that the doxorubicin conjugated to the nanoparticles reduced the amount of drug required to achieve the IC50 value with respect to free doxorubicin treatment, suggesting that the nanoformulation had an enhancement effect. The higher cytotoxic activity of the DOX nanoformulation when compared with the free DOX, especially in the range of higher drug concentrations, is presumably due to selective pH stimuli release of the drug and the accumulation within tumor cells.

In order to evaluate the impact of the nanoformulation on the required dose of the drug to achieve the therapeutic effect, the effect on cell viability of the treatment with DOX-NPs (**7**) compared to a similar concentration of free doxorubicin was measured. In particular, cells were treated with a dose of 40,000 NPs/cell of DOX-NPs (**7**) corresponding to a concentration of 500 nM of doxorubicin. As seen in Figure 3c, DOX-NPs (**7**) showed a greater cytotoxicity than free doxorubicin under cell culture conditions, the results being statistically significant with a *p*-value of <0.0001. These results show that the release of the doxorubicin conjugated to the nanoparticles was sufficient to successfully inhibit the cell proliferation of the three cancer cell lines tested. DOX-NPs (**7**) showed a therapeutic efficiency twice that of free doxorubicin, with results comparable to other nanoformulations [11,13]. This fact suggests that this nanodevice is a promising tool for the selective release of drugs.

### 3.4. Analysis of DOX-NPs (**7**)-Induced Genotoxic Effect in Cancer Cells

**Figure 4:**
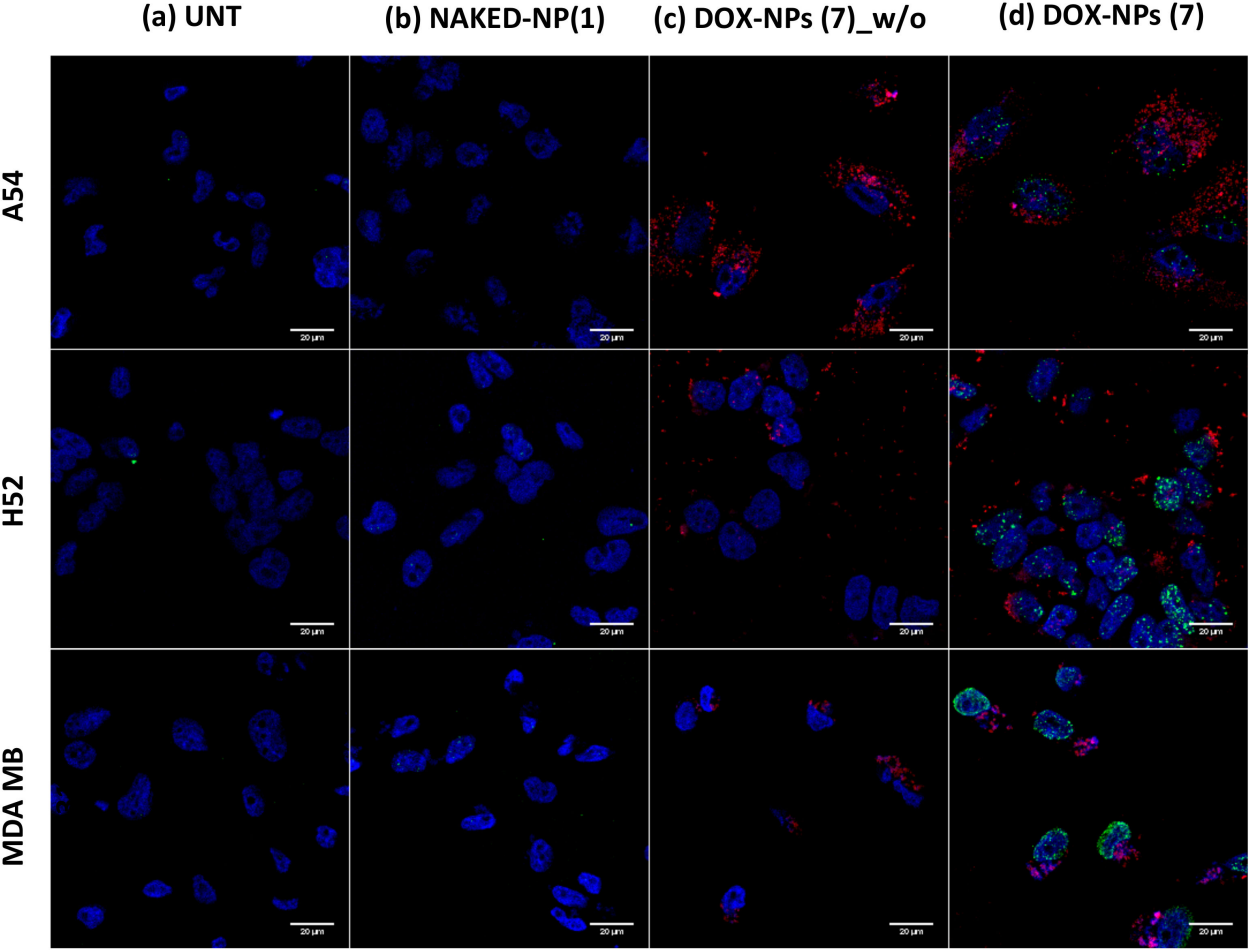
Evaluation of the DNA damage caused by the treatment with DOX-NPs (**7**) by immunostaining of γ-H2Ax in A549, H520, and MDA MB 231 cancer cell lines analyzed by confocal microcopy. The untreated cells (UNT) (**a**) and cells treated with NAKED-NPs (1) (**b**) were used as negative controls. Cells treated with DOX-NPs (**7**) were analyzed without (**c**) and with (**d**) a primary anti-γ-H2Ax antibody. Images with an increase of 63x show a composition of the four channels used: blue, DAPI for the nucleus staining; red, DOX-NPs (**7**); and green, for H2Ax staining with Alexa488-antiphospho-H2Ax.

Two mechanisms of action have been proposed by which doxorubicin acts in cancer cells: (i) intercalation in DNA and disruption of DNA repair; and (ii) generation of free radicals with subsequent damage in cell membranes, DNA, and proteins [34]. However, the confirmation of a genotoxic effect of these DOX-NPs (**7**) nanoformulations loaded with DOX in cancer cells confirms the in situ cytoplasmic release of the drug from the nanoparticle, and confirms that it reaches the nucleus; thus, the efficacy of the pH release strategy is corroborated. The genotoxic effect of the DOX conjugated to NPs (DOX-NPs (**7**)) was evaluated in the cell models studied, focusing on the damage caused in the DNA through detection of the phosphorylated form of the variant histone H2AX (γ-H2AX). The cell responds to DNA damage through the phosphorylation of thousands of H2AX molecules flanking the damaged site. This highly amplified response can be visualized as a γ-H2AX focus in the chromatin that can be detected in situ with the appropriate antibody (antiphospho-H2A.X.) using confocal microscopy analysis [28]. For this purpose, DNA damage in MDA-M-231, H520, and A549 cancer cells was determined by immunostaining of phospho-H2A.X foci (Figure 4). Untreated cells (UNT) and cells treated with NAKED-NPs (**1**) were used as negative controls for this experiment. As expected, DNA damage was not observed when cells were not treated or treated with nanoparticles without the drug being loaded (Figure 4a,b). Conversely, cells treated with DOX-NPs (**7**) but without primary staining with γ-H2Ax were analyzed to confirm the specificity of this assay. Noteworthy nanoparticles were detected (red dots) but no signal coming from unspecific immunostaining was observed (Figure 4c). In addition, the obtained results of specific immunostaining with γ-H2Ax following the treatment of the cells with DOX-NPs (**7**) show that DNA damage can be clearly observed following staining with a secondary antibody for the green channel (green foci, Figure 4d). This result corroborated the efficiency of the pH-sensitive release strategy of the drug from the nanoparticles, since the drug must be released cytoplasmically so that it is possible to enter the nucleus where DOX-NP (**7**)-induced DNA damage occurs.

## 4. Conclusions

In conclusion, a covalent strategy for the development of a PEGylated therapeutic nanosystem for pH-sensitive release was developed. Better anti-tumor activity was shown in cells treated with the nanoparticles than free DOX. The IC50 value was reduced by half by the conjugation of doxorubicin to the nanoparticle, compared to free doxorubicin, demonstrating the therapeutic capacity of DOX-NPs (**7**). This nanodevice has a proven capability of releasing the drug in a controlled manner at acidic pH and improving the drug therapeutic index by increasing efficacy compared to doxorubicin in solution. In addition, it was demonstrated to be stable for up to 6 months at 4 °C and 25 °C. The next step will involve conjugating other drugs in clinical use to this nanodevice using the same chemical strategy; thereafter, applying this therapeutic strategy to other types of cancer; and in the long term, to other pathologies. Future work will be focused on in vitro and in vivo preclinical characterization to move this nanodevice closer to clinical use.

## Supplementary Materials

The following are available online at https://www.mdpi.com/2073-4360/12/6/1265/s1, supplementary figures: Figure S1. Calibration standard curve of concentration of nanoparticles (OD 600) by spectrophometry. Figure S2. Numbers of NPs per mL calculation. Figure S3. Calibration standard curve of doxorubicin solution (OD 480) by spectrophometry. Figure S4. Calibration standard curve of doxorubicin solution by HPLC. Figure S5. Dose–response curves (percentage of cell viability versus concentration) of treatment with DOX-NPs (**7**) in the cell models studied, represented in NPs/Cell. Figure S6. Dose–response curves (percentage of cell viability versus concentration) of treatment with free doxorobucin in the cell models studied. General protocol for cellular nanofection.
