# Supplementary material for: Characterization and Therapeutic Effect of a pH Stimuli Responsive Polymeric Nanoformulation for Controlled Drug Release
Source: Polymers (Basel). 2020 Jun 1;12(6):1265. doi: 10.3390/polym12061265 (PMC7361709; doi:10.3390/polym12061265)
Supplement: Supplementary file 1 [file polymers-12-01265-s001.pdf]

Supporting Information

Characterization and Therapeutic Effect of a pH Stimuli Responsive Polymeric Nanoformulation for Controlled Drug Release

María Victoria Cano-Cortés[1,2,3], José Antonio Laz-Ruiz[1,2,3], Juan José Díaz-Mochón[1,2,3] and Rosario M. Sánchez-Martín[1,2,3]*

Supplementary figures

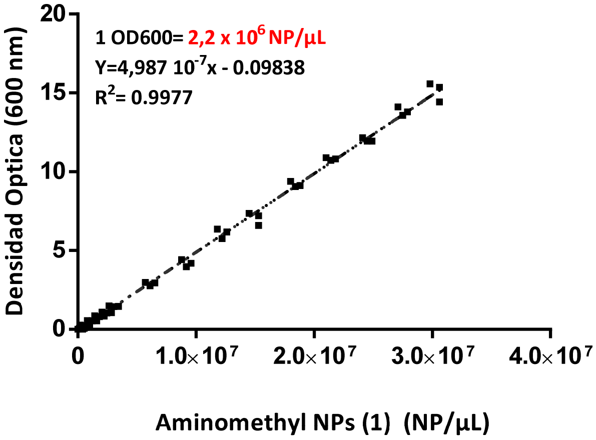

Figure S1. Calibration standard curve of concentration of nanoparticles (OD 600) by spectrophotometry.

$$Number\ of\ NPs\ per\ mL = \frac{6x10^{10} x\ Solid\ content\ (\%) x\ \rho_{suspension}}{\pi\ x\ \rho_{particles}\ x\ d^3}$$

Where: Solid content (%) = weight (%) (g per 100 mL)

$$\rho_{suspension} = \frac{100\ x\ \rho_{particles}}{Solid\ content\ (\%)\ x\ (1 - \rho_{particles}) + (100\ x\ \rho_{particles})}$$

$\rho_{particles}$ = 1 g/ dm^3 for polystyrene particles

Figure S2. Number of NPs per mL calculation (Ref. **Bangs, L-B. Uniform latex particles. 1984, Seragen Diagnostics Incorporated Ed.)**

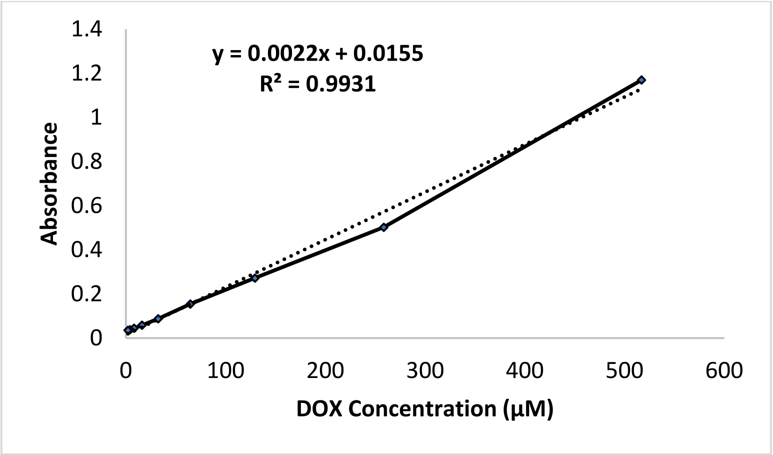

Figure S3. Calibration standard curve of free doxorubicin (OD 480) by spectrophotometry.

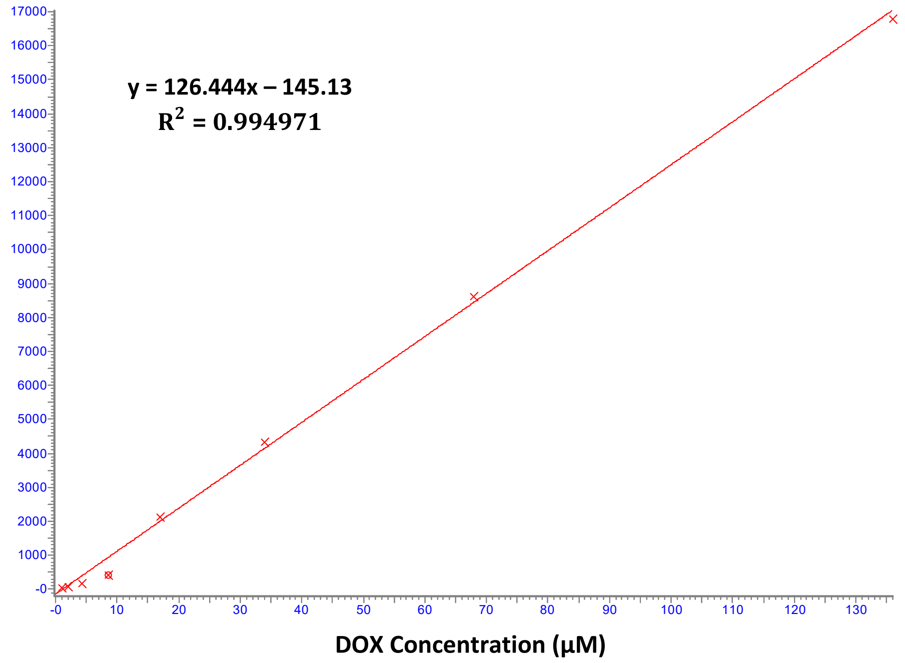

Figure S4. Calibration standard curve of free doxorubicin by HPLC.

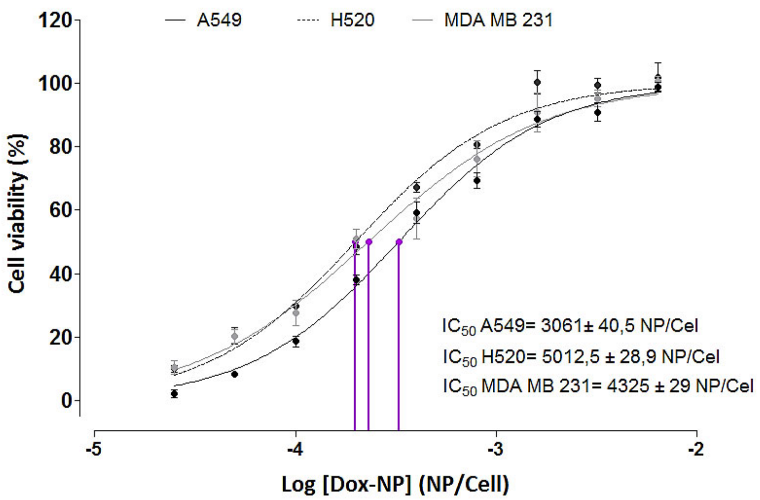

Figure S5. Dose-response curves (percentage of cell viability versus concentration) of treatment with DOX-NPs (**7**) in the cell models studied, represented in NPs/Cell.

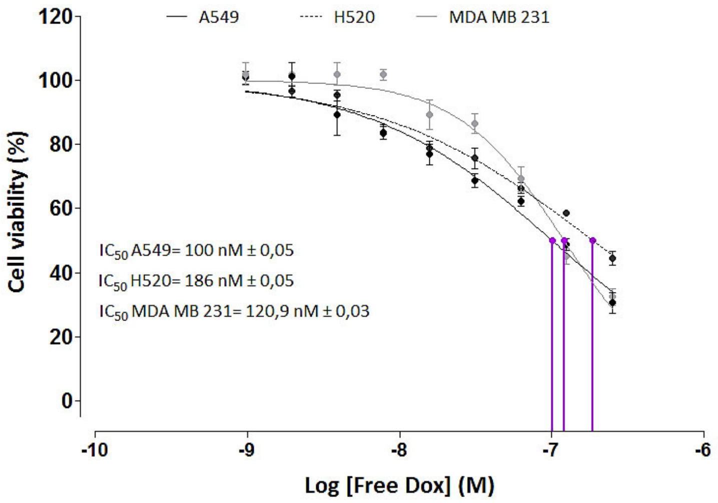

Figure S6. Dose-response curves (percentage of cell viability versus concentration) of treatment with free Doxorobucin in the cell models studied, represented in M.

.

General protocol for cellular nanofection

1. Cellular nanofection by flow cytometry

After incubation with NPs, the medium was aspirated and the cells were washed with 1xPBS and separated with trypsin-EDTA at 37 °C for 5 minutes. Then, each sample was fixed in 2% paraformaldehyde (PFA) at room temperature for 10 minutes and protected from light. Samples were analyzed by flow cytometry with a FACSCanto II flow cytometer. Each experiment was performed in duplicate by ratio and time of incubation and was repeated at least three times.

The study of the nanofection of DOX-NPs (7) was carried out using the following ratio of NPs per cell: 1/50; 1/100; 1/250; 1/500; 1/750; 1/1000; 1/2500; 1/5000; 1/7500; 1/10000. The dot plots and the cytometry stadistics were obtained using the FlowJo software. Graphs and statistical difference data were made using the GraphPad software according to the following explanation. The percentages of cell data containing NPs were plotted against the cell / NPs ratio in two different chart types. First in an XY representation according to the adjustment model of the hyperbola equation to study the saturation profile NPs of the MDA MB 231 cell line and the specific multiplicity of fifty nanofection (MNF50 index, number of NPs to obtain the 50 % of the cells that contain NPs (nanofected)). Second in a bar representation to establish the statistically significant differences mediated by the analysis of the variance of a factor (ANOVA) using the Bonferroni multiple comparison comparing the same treatments between different NPs. In addition, the median fluorescence intensity (MFI) was analyzed comprehensively by comparing the increase of the MFI (ΔMFI, MFI sample / MFI without treatment). The ANOVA multiple comparison test was also used.

2. Cellular nanofection by confocal microscopy

The cells were washed with 1X PBS, separated with trypsin / EDTA, counted and diluted with the corresponding media to a final concentration of 10 5 cells per mL. 500 μL of each cell line suspension was seeded onto glass coverslips coated with poly-L-lysine in 24-well plates and incubated for 15 h. Then, the media was replaced by a new solution with the medium containing the quantity of NPs corresponding to each experiment. After the corresponding incubation time, the medium was aspirated and the cells were washed with 1X PBS and fixed in 4% paraformaldehyde at room temperature for 30 minutes. The fixed cells were washed with 1X PBS and mounted with ProLong Gold mounting medium with DAPI (Life technologies). The images were collected with the ZEISS LSM 710 confocal laser microscope using a DIC Plan-

Apochromat 63x oil immersion objective with 1.40 numerical aperture and the ZEN 2010 software. The image analysis was subsequently carried out with the ZEN 2012 program Blue Edition or the program ImageJ version 1.49b (free software). Samples containing fluorescent NPs and DAPI nuclear staining were excited using a HeNe laser line of 633 nm wavelength (5.0 mW) and 7% power for the NPs, a laser line of 405 nm diode wavelength (30.0 mW) 2.8% power for DAPI and HeNe laser line with wavelength of 543 nm (1.2 mW) 20% power for DIC images and 1.00 Airy unit (AU). Each experiment was performed in duplicate and repeated three times per cell line.
